# Genomic insights and functional evaluation of *Lacticaseibacillus paracasei* EG005: a promising probiotic with enhanced antioxidant activity

**DOI:** 10.3389/fmicb.2024.1477152

**Published:** 2024-10-14

**Authors:** Jisu Kim, Jinchul Jo, Seoae Cho, Heebal Kim

**Affiliations:** ^1^Department of Agricultural Biotechnology, Research Institute of Agriculture and Life Sciences, Seoul National University, Seoul, Republic of Korea; ^2^eGnome Inc., Seoul, Republic of Korea; ^3^Interdisciplinary Program in Bioinformatics, Seoul National University, Seoul, Republic of Korea

**Keywords:** lactic acid bacteria, *Lacticaseibacillus paracasei*, superoxide dismutase (SOD), probiotics, antioxidant activity, SOD overexpression, inflammatory bowel disease

## Abstract

**Introduction:**

Probiotics, such as *Lacticaseibacillus paracasei* EG005, are gaining attention for their health benefits, particularly in reducing oxidative stress. The goal of this study was to reinforce the antioxidant capacity of EG005, along with comprehensive genomic analysis, with a focus on assessing superoxide dismutase (SOD) activity, acid resistance and bile tolerance, and safety.

**Methods:**

EG005 was screened for SOD activity and change of SOD activity was tested under various pH conditions. Its survival rates were assessed in acidic (pH 2.5) and bile salt (0.3%) conditions and the antibiotic MIC test and hemolysis test were performed to evaluate safety. Genetic analyses including functional identification and phylogenetic tree construction were performed. The SOD overexpression system was constructed using P_tuf_, P_ldh1_, P_lhd2_, and P_ldh3_ strong promoters.

**Results:**

EG005 demonstrated higher SOD activity compared to *Lacticaseibacillus rhamnosus* GG, with optimal activity at pH 7.0. It showed significant acid and bile tolerance, with survival rates recovering to 100% after 3 h in acidic conditions. Phylogenetic analysis confirmed that EG005 is closely related to other *L. paracasei* strains with ANI values above 98%. Overexpression of SOD using the P_tuf_ promoter resulted in a two-fold increase in activity compared to the controls. Additionally, EG005 exhibited no hemolytic activity and showed antibiotic susceptibility within safe limits.

**Discussion:**

Our findings highlight EG005’s potential as a probiotic with robust antioxidant activity and high tolerance to gastrointestinal conditions. Its unique genetic profile and enhanced SOD activity through strong promoter support its application in probiotic therapies and functional foods. Further research should be investigated to find the *in vivo* effects of EG005 on gut health and oxidative stress reduction. In addition, attB and attP-based recombination, combined with CRISPR-Cas9 technologies, could offer a more stable alternative for long-term *sodA* gene expression in commercial and medical applications.

## 1 Introduction

*Lactobacillus* is a genus of gram-positive, facultatively anaerobic, and non-spore-producing bacteria commonly found in fermented dairy foods or gastrointestinal tracts of humans and animals (Hammes and Vogel, 1995). Several *lactobacillus* species are known for their probiotic properties and have been used for food fermentation and preservation for centuries, examples including yogurt, cheese (Plessas et al., 2012), kefir (Zheng et al., 2013), sauerkraut (Yu et al., 2013), and kimchi (Lee et al., 2011a). Nowadays, they are widely known to be effective in improving digestion (Kim and Gilliland, 1983), promoting intestinal motility, strengthening immunity, and treating human diseases such as inflammatory bowel disease (IBD) (Saez-Lara et al., 2015; Justino et al., 2015; Huang et al., 2022).

*Lactobacillus* species are capable of producing and secreting a wide range of proteins essential to their survival and interaction with their environment (Lee et al., 2011b). These proteins include enzymes (Danilova and Sharipova, 2020), bacteriocins (Nes et al., 2001), and other bioactive molecules (Griffiths and Tellez, 2013) that contribute to their beneficial effects. In this study, we specifically focused on antioxidant capacity, as it critically involves in resisting oxidative stress and significantly impacts survival (Chooruk et al., 2017).

Naturally formed during cellular metabolism, reactive oxygen species (ROS) are chemically reactive molecules originating from oxygen molecules (Bayr, 2005). ROS can damage cell membranes, proteins, and DNA, causing oxidative stress that can induce various diseases such as IBD (Patlevič et al., 2016). This is why we focused on the antioxidant activity of lactic acid bacteria. More specifically, *Lactobacillus* can produce various antioxidants, that can scavenge free radicals and protect cells from oxidative stress (Suzuki et al., 2013). In antioxidants, *Lactobacillus* produces SOD, which is classified into Mn-SOD, Fe-SOD, Cu-SOD, and Zn-SOD based on the metal cofactor presented in the active site of the enzyme. SOD is derived from species such as *Lactobacillus acidophilus*, *Lacticaseibacillus casei*, and *Lacticaseibacillus rhamnosus*, and is effective in alleviating oxidative stress in inflammatory bowel disease (IBD) when delivered to the intestines (LeBlanc et al., 2011). Additionally, SOD helps reduce oxidative stress during storage, thereby enhancing the shelf life and maintaining the quality of foods (Arasu et al., 2015). Using *lactobacillus* strains as a natural antioxidant source is gaining attention in the food and nutraceutical industries due to their potential health benefits (Ghiasi et al., 2023; Khubber et al., 2022).

Although many species of lactic acid bacteria with potent probiotic characteristics have been identified through extensive research, they are still insufficient to meet the growing demands of both humans needs and the expanding scale of industry. In this study, we found that strain EG005 exhibited relatively higher SOD activity compared to other strains in our laboratory. To determine the probiotic properties of EG005, the enzyme characterization of SOD in EG005, acid and bile resistance, antibiotic MIC tests, and hemolysis tests were performed. The genome characteristics, phylogenetic analysis of EG005, and the evolutionary patterns of the superoxide dismutase gene (*sodA*) among other *Lacticaseibacillus paracasei* strains were also identified. Additionally, the *sodA* gene from the EG005 genome was cloned with four selected strong promoters, and the resulting plasmid vectors for *sodA* overexpression were then transformed into EG005. Finally, each overexpressed SOD activities were compared in response to oxidative stress.

This research focuses on investigating the beneficial properties of *L. paracasei* EG005 as a probiotic through safety evaluation experiments and genomic analysis, with a particular emphasis on its superoxide dismutase (SOD) activity along with the overexpression of the antioxidant capability. We aim to determine whether antioxidant capacity not only enhances survival in a harsh gastrointestinal environment but also contributes to reducing oxidative stress.

## 2 Materials and methods

### 2.1 Sample collection and bacterial species

In this study, EG005, which was isolated from fermented cheese from a local market in Gwanak, Seoul, and previously identified as *L. paracasei,* was used (Jeon et al., 2022). It was stored in de Man, Rogosa, and Sharpe (MRS) medium (Difco, USA) containing 25% glycerol at −80°C. The *E.coli* DH5α (New England Biolabs, USA) strain was used for gene cloning and plasmid propagation. *E.coli* DH5α transformants were grown in Luria-Bertani (LB) medium (Difco, USA) at 37°C in a shaking incubator during overnight (O/N). The medium was supplemented with 100 μg/mL of ampicillin for *E. coli* and 15 μg/mL of erythromycin for *L. paracasei* to select transformed colonies.

### 2.2 Antioxidant activity screening

#### 2.2.1 Antioxidant activities of LAB

Various *Lactobacillus* species were grown on the MRS agar plates in an anaerobic container at 37°C for 2 days. A single colony of each *Lactobacillus* species was inoculated in 5 mL of MRS broth medium and cultured overnight at 37°C. After incubation, the intact cells and supernatant were separated by centrifugation at 13,000 rpm for 2 min at 4°C. The cells were washed three times with Phosphate-Buffered Saline (PBS) and resuspended in PBS to adjust the cell amount to 1.0 × 10^10^ CFU/mL. The cells were disrupted by sonication (Power Sonic 405, Hwashin, Korea) at 40 kHz for 20 min. These cell debris were removed by centrifugation at 13,000 rpm for 10 min at 4°C and the cell-free extracts (CFEs) were obtained for water-soluble tetrazolium 1 (WST-1) assay (Peskin and winterbourn, 2000).

The SOD activity of *Lactobacillus* species was analyzed by using the EZ-SOD assay kit (Dogen Bio Co. Ltd., Korea). A total 20 μL of CFEs were mixed with 200 μL of WST working solution and 20 μL of Xanthine oxidase working solution, then the mixture was incubated at 37°C for 20 min. Absorbance at 450 nm was measured by using a spectrophotometer.

#### 2.2.2 Effect of pH on SOD in EG005

To characterize the effects of pH on SOD activity, measurements were taken across various pH conditions, ranging from pH 3 to 10 (Pinmanee et al., 2023). Buffer solutions used for each pH were as follows: sodium acetate buffer for pH 3.0–5.0, Tris–HCl buffer for pH 6.0–10.0, and PBS for pH 7.0. The experiments were performed in triplicate.

### 2.3 Evaluation of acid and bile tolerance of EG005

#### 2.3.1 Acid tolerance evaluation

The acid tolerance test of EG005 was performed according to the method of Conway et al. (1987), with minor modifications. A total 1 mL of overnight broth culture was inoculated into 9 mL of MRS broth adjusted to pH 2.5 using 1 M HCl and incubated at 37°C for 3 h. Samples were taken at time intervals and were 10-fold serial diluted with sterilized PBS pH 7.4 in 1.7 mL microcentrifuge tubes. A total 100 μL of diluted samples were plated on MRS agar plates. Then plates were incubated under anaerobic conditions at 37°C for 48 h. The survival rate was calculated as follows:


$$\mathrm{Survival}\;\mathrm{rate}\;\left(\%\right)=\frac{CFU/\mathrm{mL}\;\mathrm{at}\;\mathrm{specific}\;\mathrm{time}\;\left(C_{t}\right)}{CFU/\mathrm{mL}\;\mathrm{at}\;\mathrm{initial}\;ime\;\left(C_{0}\right)}\times 100$$

#### 2.3.2 Bile tolerance evaluation

The bile tolerance of EG005 was evaluated according to the method of Hassanzadazar et al. (2012), with minor modifications. The bile solution was prepared by adding powdered oxgall (Difco, USA) to MRS broth to reach a final concentration of 0.3%. A total 10 mL of cultures, consisting of 1 mL of overnight broth culture and 9 mL of medium, were incubated at 37°C for 6 h and were taken every hour to calculate the survival rate. Serial dilution and survival rate calculations were carried out in the same way as the acid tolerance experiment previously described.

### 2.4 Safety assessment

#### 2.4.1 Hemolytic activity

To evaluate the potential hemolytic activity of EG005, the overnight broth culture of EG005 and *Staphylococcus aureus* which was used as positive control were streaked on sheep blood agar plates (MBcell, KisanBio, Korea) and incubated at 37°C for 48 h, under aerobic conditions. Subsequently, the plates were visually inspected for hemolytic activity, characterized by the formation of clear zones around the bacterial colonies. Hemolytic patterns were classified as *α*-hemolysis (green zones), ß-hemolysis (clear zones), or *γ*-hemolysis (no zones) (Mangia et al., 2019).

#### 2.4.2 Antibiotic resistance assessment

For the antibiotic resistance test, the minimal inhibitory concentration (MIC) of each antibiotic for EG005 was investigated on the European Food Safety Authority (EFSA) recommendations (Additives and Feed, 2012). A total of eight antibiotics (ampicillin, gentamycin, kanamycin, streptomycin, erythromycin, clindamycin, tetracycline, chloramphenicol) with concentrations ranging from 0.25 to 256 ug/mL were used. A single colony of EG005 was inoculated into 5 mL of MRS broth medium and cultured at 37°C for 18 h. The MIC test was performed using the broth microdilution method (Wiegand et al., 2008). First, 90 uL of broth medium composed of Iso-Sensitest (MBcell, KisanBio, Korea) and MRS at a 9:1 ratio (Klare et al., 2005) was dispensed into a 96-well plate. Then, 10 ul of antibiotic solution at each concentration was added to each well so that the final antibiotic concentration of the medium was 0.25–256 ug/mL. The strain diluted with PBS at a concentration of 5.0 × 10^6^ CFU/mL was inoculated into each well at 10 uL to achieve a final concentration of 5.0 × 10^4^ CFU/well. The inoculated medium was cultured anaerobically at 37°C for 48 h. The MIC was established through visual observation, identifying the concentration at which antibiotics suppressed bacterial growth by 80% or more relative to the control.

### 2.5 Genome characterization

#### 2.5.1 Genome assembly and annotation

The raw read data of EG005 was obtained from the Sequence Read Archive (SRA, SRA run accession: SRR16961897) (Leinonen et al., 2010) deposit in the National Center for Biotechnology Information (NCBI) database. Adapter trimming was performed using Porechop v0.2.4.^1^ Genome *de novo* assembly was implemented using Canu v2.2 with options of “corMhapOptions = −threshold 0.5 −ordered-sketch-size 1,000 −ordered-kmer-size 14” and “corErrorRate = 0.105” (Koren et al., 2017). Then, the starting point of the genome was rearranged by the fixstart plugin from Circlator v1.5.5 (Hunt et al., 2015). Medaka v1.7.1^2^ with the r941_min_hac_g507 model and Homopolish v0.4.1 (Huang et al., 2021) with R9.4.pkl were utilized to convert the draft assembly genome into a higher-quality genome. The quality of the genome assembly was assessed using BUSCO v5.4.2 with the lactobacillales_odb10 dataset (Manni et al., 2021). Gene annotation of EG005 was determined by Prokka v1.14.6 (Seemann, 2014) with the -rfam option.

#### 2.5.2 Identification of gene function and safety analysis

For the Identification of gene functions, Kyoto Encyclopedia of Genes and Genomes (KEGG) and Cluster of Orthologous Groups of Proteins (COG) (Galperin et al., 2021) analyses were performed using the eggnog-mapper v2.1.12 (Cantalapiedra et al., 2021) with a 90% identity threshold and an E < 10^−3^. The antibiotic resistance genes and virulence factors in the genome of EG005 were verified using ABRicate v1.0.1^3^ to determine its safety as a probiotic.

#### 2.5.3 Phylogenetic analysis

CVtree was performed using a composition vector (CV) matrix with *k* = 6 parameters on nucleotide sequences for the whole-genome-based phylogenetic tree (Qi et al., 2004). The evolutionary aspects of EG005 were investigated by analyzing 63 whole genome sequences, including 61 other *L. paracasei* strains retrieved from NCBI (Sayers et al., 2022) and *Bacillus subtilis* DSM10 was used as the outgroup. Additionally, to construct a *sodA* gene tree for EG005, *sodA* genes were extracted from the same 61 *L. paracasei* strains used above. Then, MAFFT v7.525 was used for the multiple sequence alignment (Katoh and Standley, 2013), and the tree was created using IQ-TREE v2.3.3_beta (Minh et al., 2020) with 1,000 bootstrap values (Hoang et al., 2018), employing a quick and efficient stochastic algorithm to infer phylogenetic trees by maximum likelihood (ML). For species delineation, the average nucleotide identity (ANI) was calculated using PYANI v0.2.12 with the ANIm parameter (Pritchard et al., 2016).

### 2.6 SOD overexpression in EG005

#### 2.6.1 Construction of SOD overexpression plasmids

The genomic DNA of EG005 was isolated using the G-spin Genomic DNA Extraction Kit (iNtRON Biotechnology, Korea) for bacteria, following the manufacturer’s protocol. Subsequently, DNA fragments necessary for the construction of overexpression plasmids, such as the *sodA* gene and strong promoters [*tuf* promoter (P_tuf_), *ldh1* promoter (P_ldh1_), *ldh2* promoter (P_ldh2_), and *ldh3* promoter (P_ldh3_)], were amplified via PCR. The PCR was conducted using PrimeSTAR^®^ GXL DNA polymerase (Takara, Japan) and several oligonucleotide primer sets, which are listed in Supplementary Table S1. Each PCR product was then subcloned using the pGEM-T Easy Vector Systems (Promega, USA) to obtain pJS-P_tuf_, pJS-P_ldh1_, pJS-P_ldh2_, pJS-P_ldh3_, pJS-S1, and pJS-S2. To construct pJS2-P_tuf_, pJS2-P_ldh1_, pJS2-P_ldh2_, and pJS2-P_ldh3_, the enzyme sites on pJS-S1 and pJS-S2 were digested by treatment with HindIII/EcoRV restriction enzymes for pJS-S1, and KpnI/ClaI restriction enzymes for pJS-S2, respectively. Then, each strong promoter DNA fragment was digested from pJS-P_tuf_, pJS-P_ldh1_, pJS-P_ldh2_, and pJS-P_ldh3_ by appropriate enzyme sites which are described in Supplementary Table S2. These DNA fragments harboring each strong promoter were inserted into linearized pJS-S1 or pJS-S2 using T4 DNA ligase (Thermo Fisher Scientific, USA). Consequently, four *sodA* genes conjugated with each strong promoter in pJS2-P_tuf_, pJS2-P_ldh1_, pJS2-P_ldh2_, and pJS2-P_ldh3_ were double-digested again using ApaI/EcoRV or ApaI/ClaI restriction enzymes and inserted into linearized pLEM415-ldhL-mRFP1 (Addgene plasmid # 99842) (Bao et al., 2013) to construct plemJS-P_tuf_, plemJS-P_ldh1_, plemJS-P_ldh2_, and plemJS-P_ldh3_. The recombinant plasmids were confirmed by DNA sequencing using several primers which were listed in Supplementary Table S1 (Cosmogenetech, Korea).

#### 2.6.2 Electroporation of EG005

Electroporation of EG005 was conducted as described by Mason et al. (2005) with minor modifications. A total 2 mL of overnight broth culture of EG005 was inoculated into 100 mL of MRS broth containing 2.5% glycine. The incubation was continued at 37°C, 140 rpm in a shaking incubator until the optical density at 600 nm reached a value between 0.6 and 0.8. The cells were harvested by centrifugation at 4,000 rpm for 10 min at 4°C and washed three times with ice-cold sterilized deionized water. The harvested cells were resuspended in 50 mM EDTA, incubated on ice for 5 min, and washed once in deionized water again. Then, the cells were washed twice with an electroporation buffer consisting of 0.5 M sucrose and 10% glycerol. After discarding the supernatant, the pellets were resuspended in 800 uL of electroporation buffer solution to endow EG005 with foreign DNA competency. The EG005 competent cell was mixed with 2 μg of plasmid DNA and transferred into a pre-chilled electro cuvette with a 2 mm electrode gap. Then, it was electroporated using an electroporator (Bio-Rad, USA) with a parameter of 1.8 kV pulse voltage. After the electroporation, 1 mL of MRS broth was immediately added and the suspended cells were transferred into a 1.7 mL microcentrifuge tube and subsequently incubated for 3 h at 37°C. The incubated cells were spread on an MRS agar plate containing erythromycin and incubated at 37°C for 48 h under anaerobic conditions.

## 3 Results

### 3.1 Antioxidant activity of EG005 and pH influence on SOD

**Figure 1:**
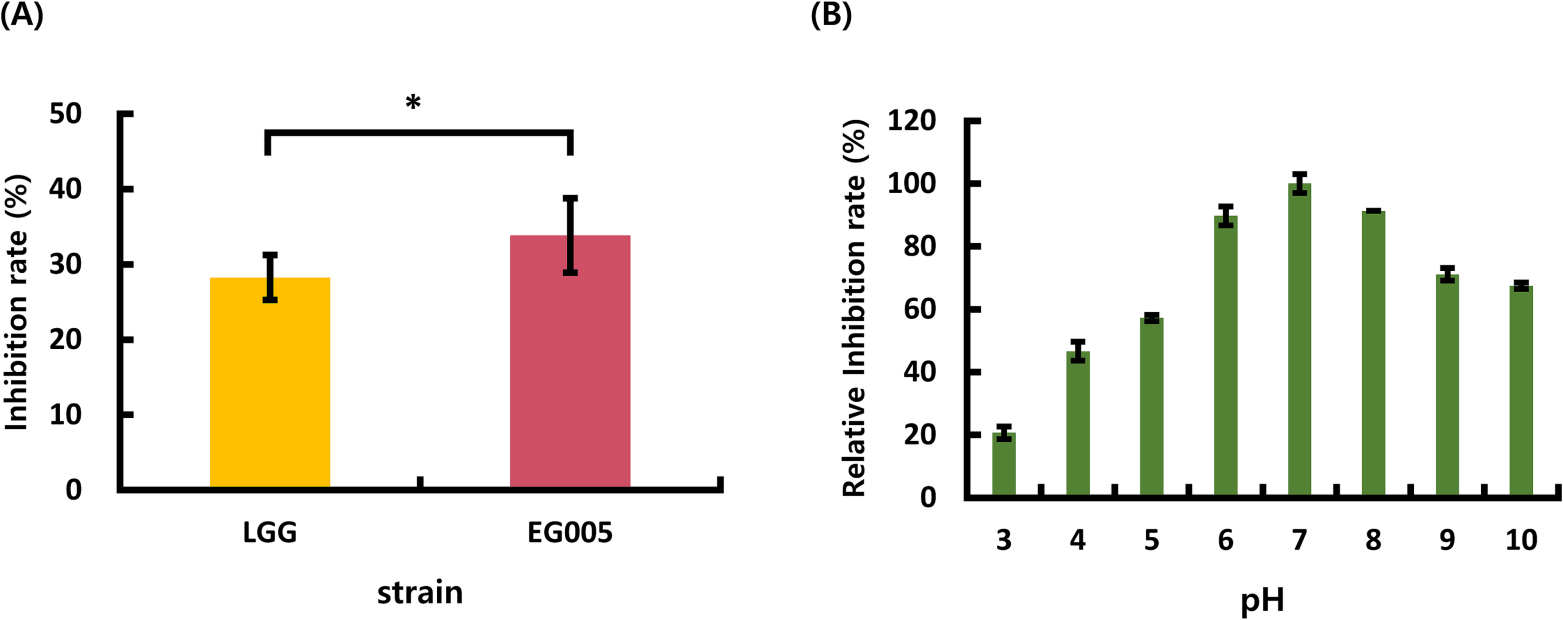
**(A)** Superoxide dismutase (SOD) activity results of *Lactobacillus rhamnosus* GG (LGG) and strain EG005. The results show that there is a statistically significant difference in the SOD inhibition rate between LGG and EG005 (*p* = 0.0339), indicating a higher antioxidant capacity in strain EG005. **(B)** The effect of pH on SOD activity in EG005. EG005 SOD showed the highest activity at pH 7. Error bars represent the standard deviation of three independent experiments.

Among the 24 strains which were isolated from several fermented foods in the previous study, EG005 showed the highest antioxidant activity through SOD activity assay (The data is not shown). Compared to *L. rhamnosus* GG (LGG), The inhibition rate for ROS formation of EG005 was 5.4% higher than that of LGG (Figure 1A). A Student’s *t*-test was conducted to analyze the difference between EG005 and LGG, revealing a statistically significant *p*-value of 0.0339. The effects of pH on EG005 SOD activity were investigated by incubating the strain under different pH conditions ranging from 3.0 to 10.0 at a constant temperature of 37°C. The result showed that the optimal activity of SOD was observed at a pH level of 7.0 (Figure 1B). The relative antioxidant activity of EG005 at pH 7.0 was five times higher than that at pH 3.0, which exhibited the lowest activity. Approximately 70% of the antioxidant activity was maintained at alkaline pH levels of 9.0 and 10.0. As a result, EG005 SOD showed relatively high antioxidant activity across a wide range of pH conditions, from neutral to alkali.

### 3.2 Survival of EG005 in acidic and bile environment

**Figure 2:**
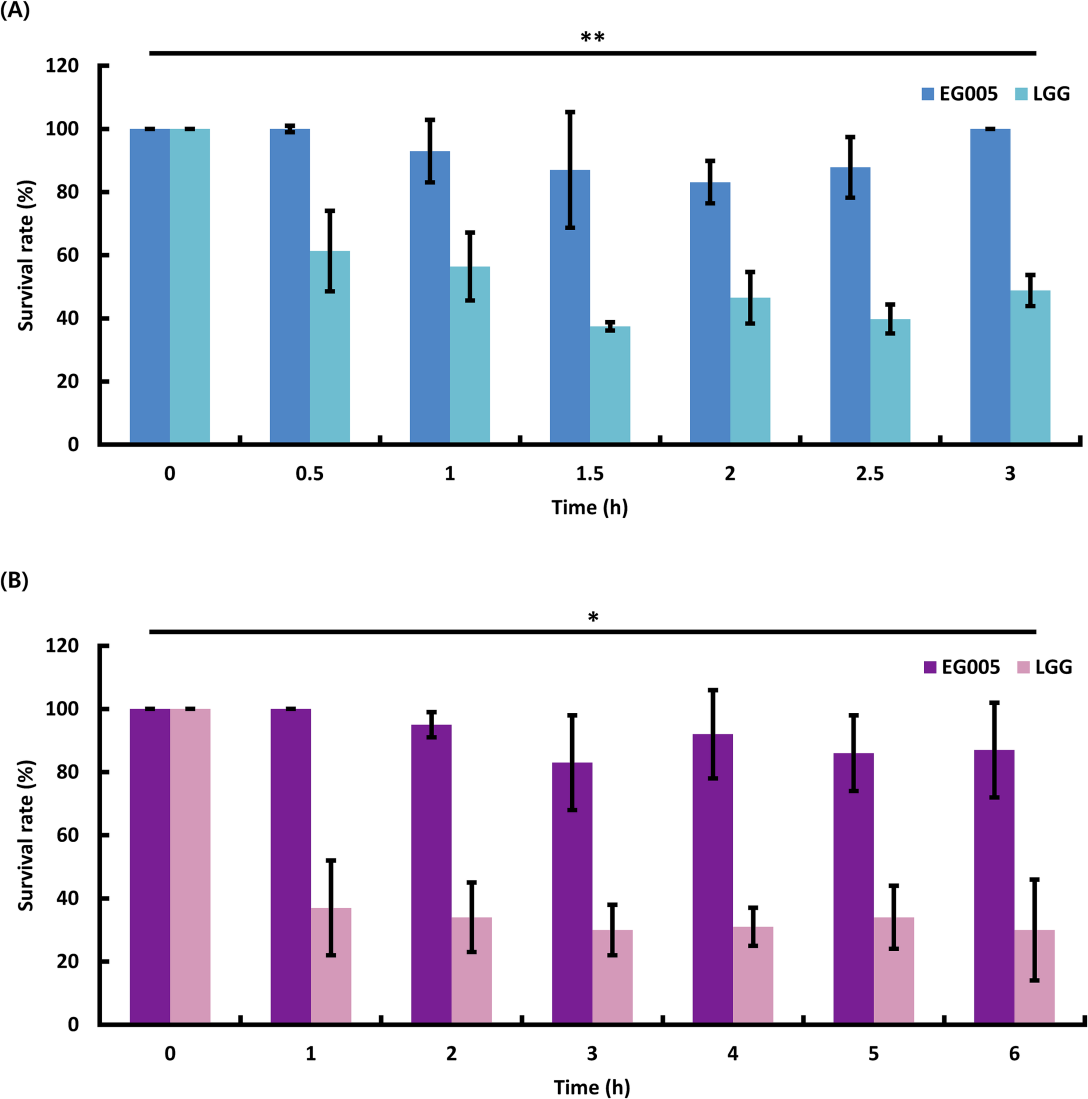
Probiotic properties of EG005. LGG was used as a control to compare the acid and bile tolerance of EG005. **(A)** The survival rate of EG005 at pH 2.5, was measured at 30-min intervals for 3 h. The results show that there is a statistically significant difference between LGG and EG005 (*p* = 0.003, **p < 0.01). **(B)** Survival rate of EG005 at 0.3% bile salt, measured at 1-h intervals for 6 h. Error bars represent the standard deviation of three independent experiments. The results show that there is a statistically significant difference between LGG and EG005 (*p* = 0.013, *p < 0.05).

To evaluate the acid tolerance of EG005 in acidic conditions, such as gastric juice, the survival rates of EG005 and LGG were compared in an acidic MRS medium adjusted to pH 2.5 for 3 h. EG005 exhibited no decrease in survival rate after the initial 30-min exposure to pH 2.5 (Figure 2A). Afterward, a slight decrease was observed until 2 h, followed by a subsequent increase in survival rate, nearly returning to the level observed at the outset (0 h). In contrast, the survival rate of LGG decreased sharply after 30 min and declined to 49% after 3 h. Overall, EG005 exhibited a significantly higher survival rate at acidic conditions than the LGG. To assess the bile tolerance of EG005, the survival rates of EG005 and LGG were compared in MRS broth containing 0.3% bile salt for 6 h. LGG showed a sharp decrease in survival rate to below 40% after 1 h, followed by a further decrease to 30% after 6 h. Conversely, EG005 maintained its survival rate for up to 1 h, followed by a gradual decline, but still maintaining a survival rate above 80% even after 6 h (Figure 2B). A linear mixed model was applied to statistically analyze the results, incorporating a time-squared term to account for the non-linear pattern of bacterial survival. The analysis revealed a *p*-value of 0.003 for acid resistance, indicating that EG005’s survival rate was significantly less affected compared to LGG. Similarly, for bile resistance, the p-value was 0.013, confirming a significant difference between the two strains.

### 3.3 Hemolytic activity and antibiotic resistance in EG005

**Figure 3:**
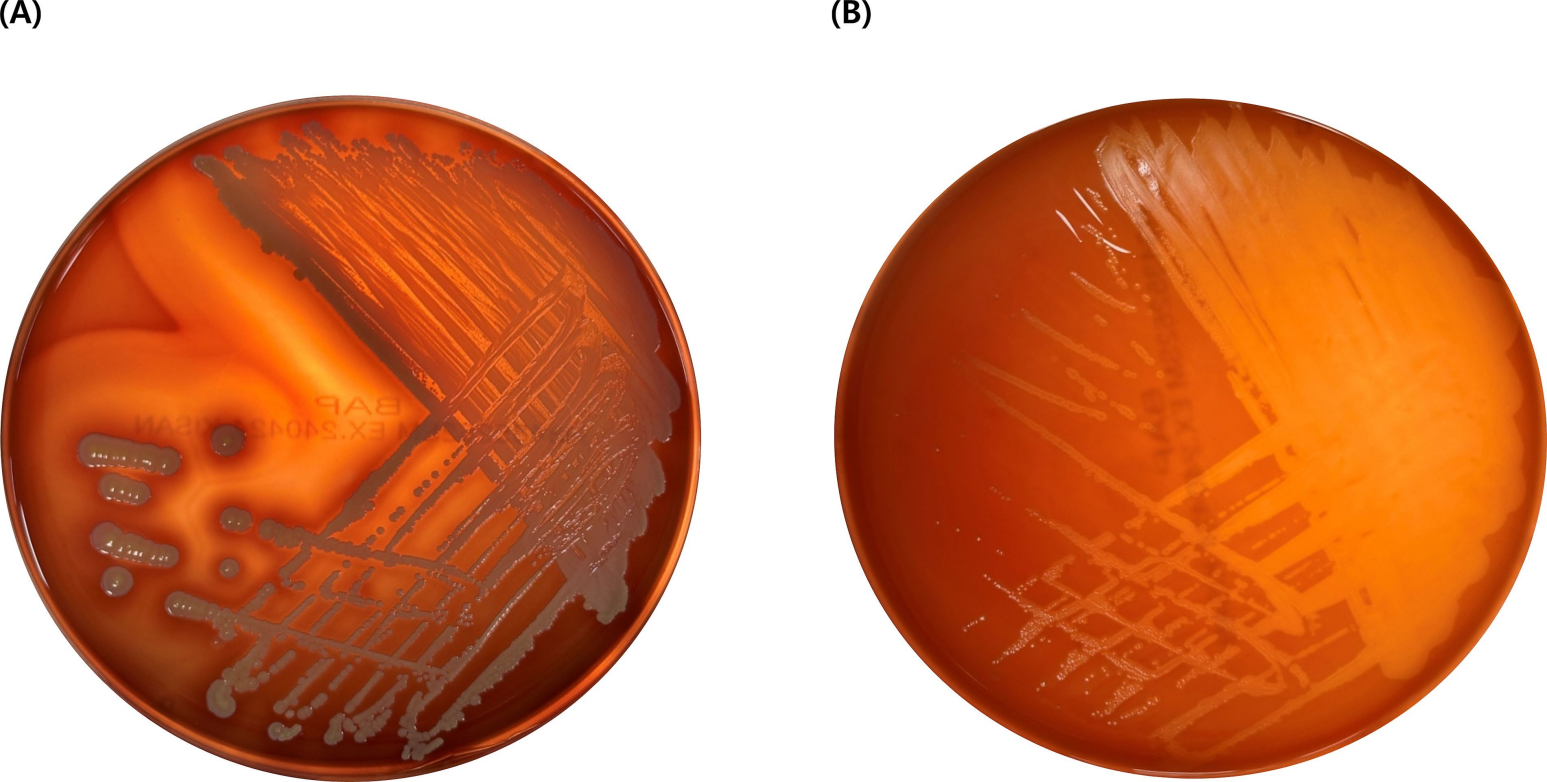
Result of hemolysis test. **(A)** *Staphylococcus aureus* exhibits *β*-hemolysis, creating a clear zone due to the complete lysis of red blood cells. **(B)** *L. paracasei* EG005 shows *γ*-hemolysis without a clear zone, indicating no lysis of red blood cells.

In the hemolytic activity test, EG005 showed neither *β*-hemolysis nor *α*-hemolysis, as it did not exhibit any clear zone on the sheep blood agar plate and there was no change around the colonies (Figure 3B). This suggested that EG005 did not possess hemolytic properties, which is a desirable trait for probiotics to ensure safety. In contrast, *S. aureus* which was used as a positive control showed clear zones indicating β-hemolysis (Figure 3A). To evaluate the antibiotic resistance of EG005, an antibiotic susceptibility test was performed according to the EFSA guidelines for probiotics. Consequently, EG005 was susceptible to most antibiotics, including ampicillin, gentamicin, streptomycin, erythromycin, clindamycin, tetracycline, and chloramphenicol, except for kanamycin (Table 1A). The susceptibility to these antibiotics met safety standards, as it indicated that EG005 lacked broad antibiotic resistance. At the genomic level, an antibiotic resistance gene analysis was conducted using ABRicate software to determine whether EG005 harbored any antibiotic-resistance genes, but none were identified (Table 1B). Additionally, no virulence genes were detected in the ABRicate results.

**Table 1:** Safety evaluation of *L. paracasei* EG005 as a probiotic. **(A)** Minimum inhibitory concentration (MIC) values of EG005 against various antibiotics. **(B)** Comparison of detected genes between EG005 and *S. aureus* MRSA252 using ABRicate across various databases. EG005 showed no detected genes, whereas *Staphylococcus aureus* MRSA252 exhibited multiple virulence factors and resistance genes.

(A)			
Antibiotic	MIC (μg/mL)	EFSA cut-off value (μg/mL)	Resistant or sensitive
Ampicillin	4.00	4	S
Gentamicin	8.00	32	S
Kanamycin	256.00	64	R
Streptomycin	32.00	64	S
Erythromycin	≤ 0.25	1	S
Clindamycin	≤ 0.25	1	S
Tetracycline	4.00	4	S
Chloramphenicol	4.00	4	S

(B)			
	Database	EG005	*S. aureus* MRSA252
Antibiotic resistance genes	Megares	0	27
	Card	0	16
	Argannot	0	15
	NCBI	0	14
	Resfinder	0	7
Virulence genes	Vfdb	0	63
	Plasmidfinder	0	4
	Ecoli_vf	0	0
	Ecoh	0	0

### 3.4 Genomic features of EG005

**Figure 4:**
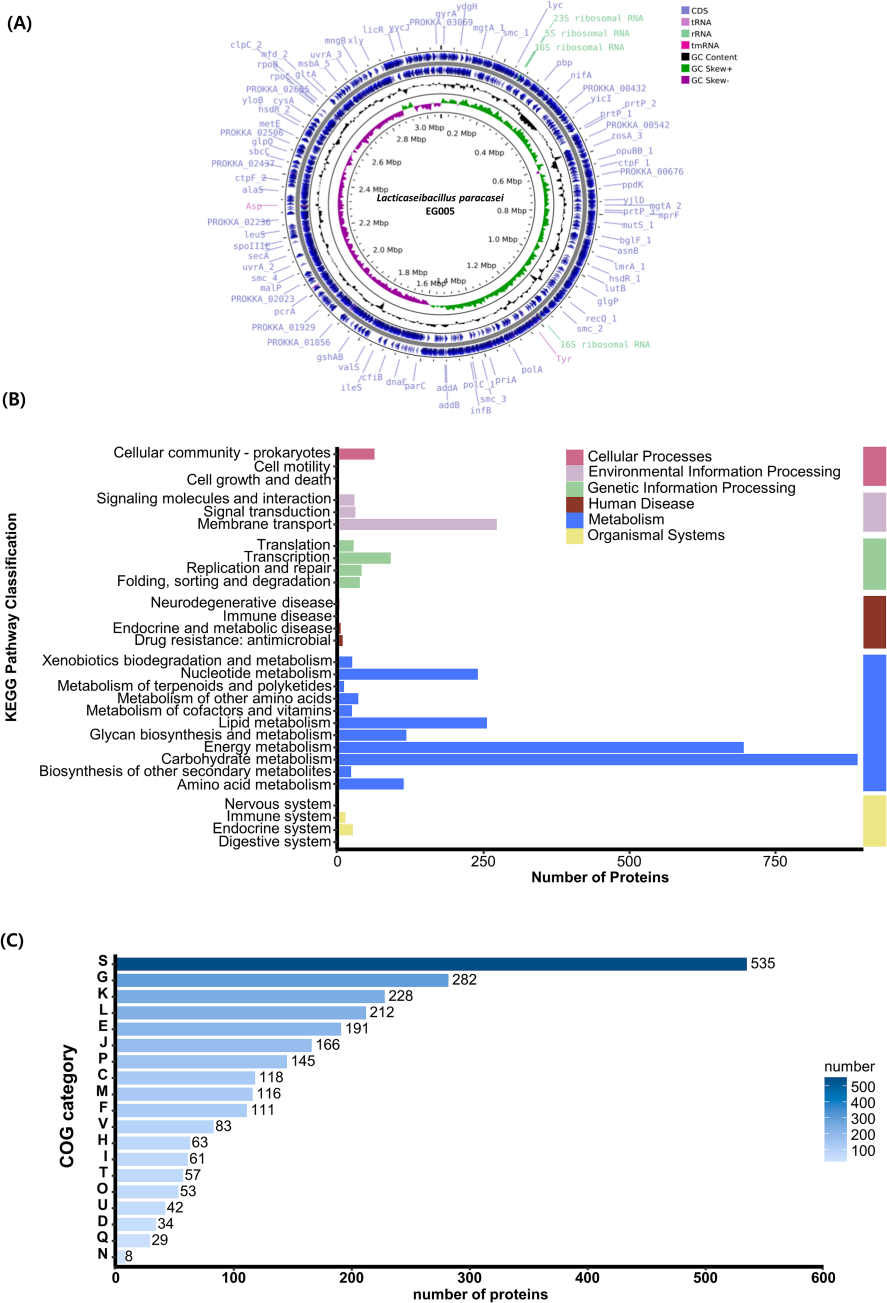
Genomic characterization of *L. paracasei* EG005. **(A)** A circular genome map of EG005, generated by CGView. From outside to inside: CDS (light blue), tRNA (light purple), rRNA (emerald green), tmRNA (magenta), GC Content (black), GC Skew+ (green), GC Skew- (purple), GC Content (black). **(B)** KEGG pathways distribution of the genes in *L. paracasei* EG005. **(C)** COG categories distribution of the genes in *L. paracasei* EG005.

A single contig was generated by performing *de novo assembly*. Following a round of polishing, a full circular genome consisting of 3,073,522 base pairs with a GC content of 46.34% was produced (Figure 4A). Prokka annotation predicted a total of 3,081 genes, including 3,006 coding sequences (CDS), 15 ribosomal RNA (rRNA) genes, 59 transfer RNA (tRNA) genes, and one transfer-messenger RNA (tmRNA) gene. The BUSCO complete value was 99.5%, indicating a highly complete and well-assembled genome with minimal missing gene content. The functional genome analysis of EG005 was analyzed by KEGG and COG databases, classifying all CDS into 29 KEGG functional categories, with “Carbohydrate metabolism” being the most prevalent, highlighting the strain’s efficiency in utilizing various carbohydrates. The next most prevalent categories were “Energy metabolism,” and “Membrane transport” (Figure 4B). Additionally, a total of 2,534 genes were classified into 19 categories in the COG function classification, including 535 genes for “Function unknown (S).” Among the COG categories, a significant number of genes (228) were assigned to the “Carbohydrate transport” and “Metabolism (G),” followed by “Transcription (K)” with 228 genes, and “Replication, recombination, and repair (L)” with 212 genes (Figure 4C). Through an additional identification of genes involved in the antioxidant activity of EG005, several related genes were discovered, including superoxide dismutase (*sodA*), NADH oxidase (*nox1, nox2*), pyruvate oxidase (*pox5*), glutathione reductase (*gshR*), thioredoxin reductase (*trxB*), nitro reductase (*nfhA*), oxidoreductase (*ydhF*), and manganese transporter (*psaA1, psaA2*), etc. (data not shown). The presence of these genes underscores the antioxidant activity of EG005, which could have contributed to its protective effects under oxidative stress conditions.

### 3.5 Phylogenetic relationships of EG005

**Figure 5:**
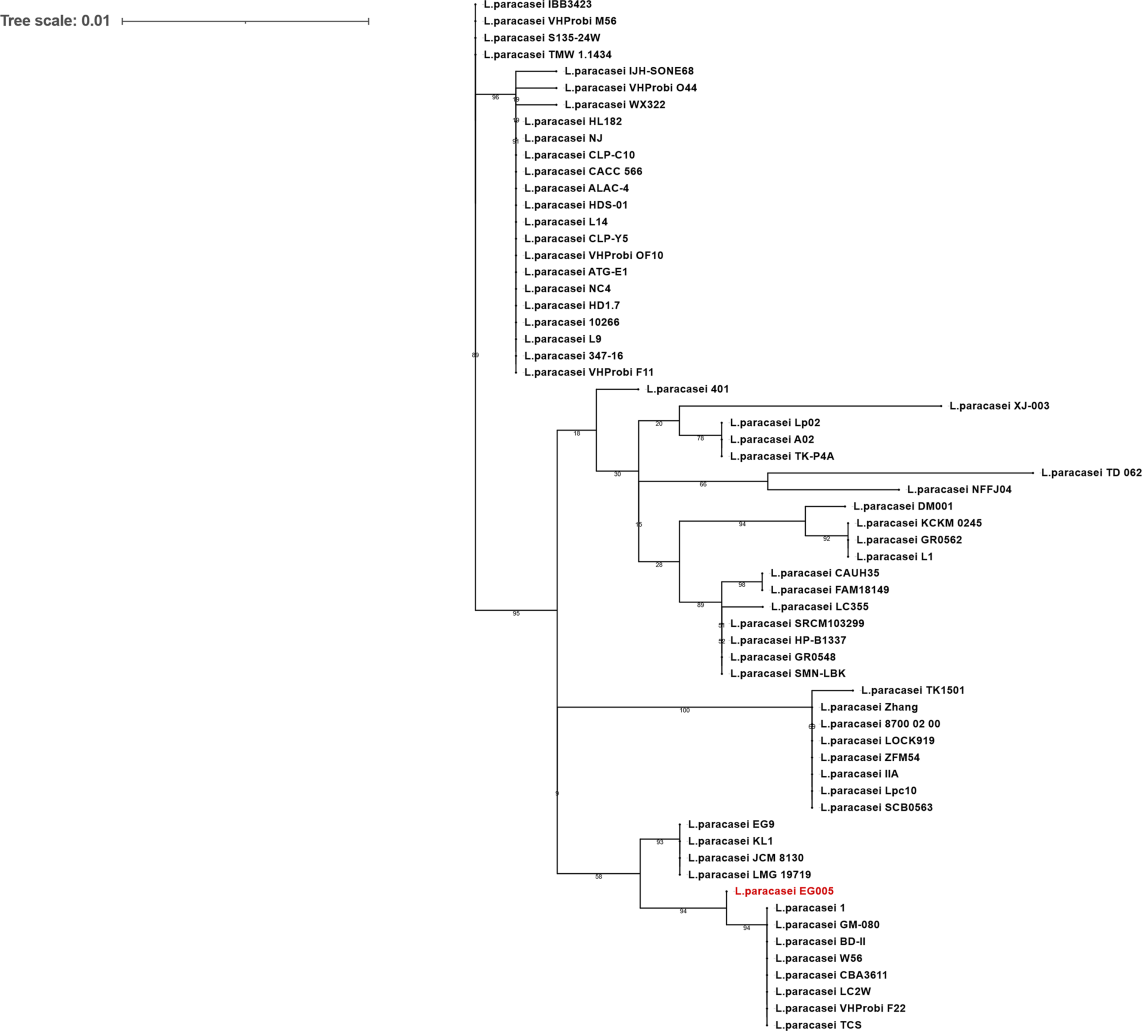
The *sodA* gene phylogeny of *L. paracasei* strains. The phylogenetic tree was constructed using the *sodA* gene sequences extracted from 62 strains of *L. paracasei* in the NCBI database. The tree reveals evolutionary relationships among *sodA* genes and EG005 appears in red. The numbers on the branches are bootstrap values with 1,000 replicates and the tree scale is 0.01.

**Figure 6:**
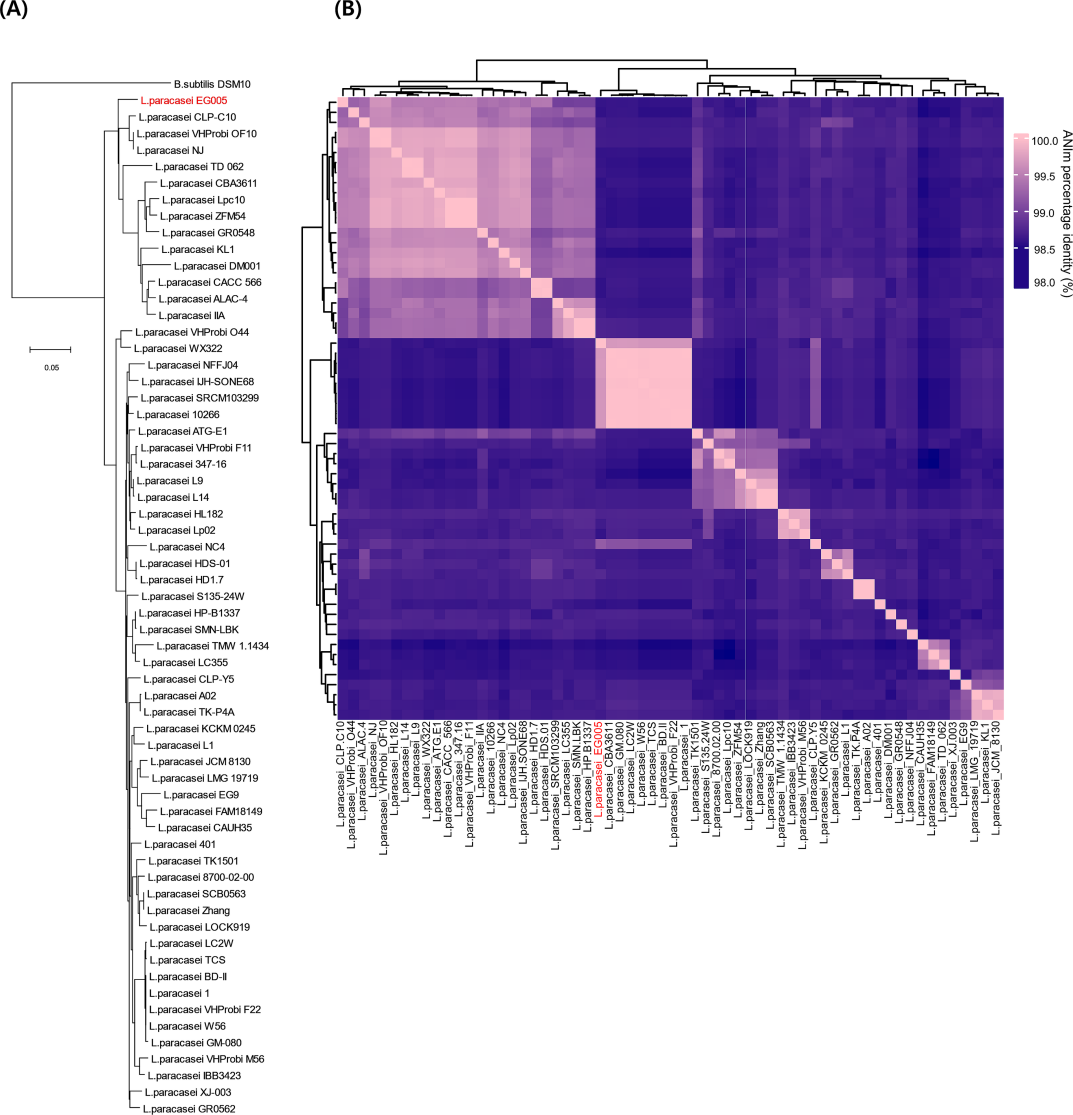
**(A)** The genetic relationships of 62 *L. paracasei* strains. The phylogenetic tree was constructed using CVTree 3.0 based on the whole-genome sequences. *L. paracasei* EG005 appears in red. *B. subtilis* DSM10 was used as an outgroup. The branch length represents overall evolutionary trends among the strains and the tree scale is 0.05. **(B)** ANI heatmap diagram showing the ANI value among 62 *L. paracasei* strains. The highest ANI value was 99.7% between EG005 and GM-080.

As described above, multiple genes related to antioxidant activity were found in the genome of EG005. Among them, the *sodA* gene encoding superoxide dismutase (SOD) was selected and a gene tree for *sodA* was constructed to assess the differences between the *sodA* genes present in 61 strains of *L. paracasei*. As a result, the *sodA* gene of EG005 formed a close cluster with those of GM-080, LC2W, CBA3611, 1, VHProbi_F22, BD-II, TCS, and W56 (Figure 5). Interestingly, despite the close clustering, none of the *sodA* gene sequences from those strains were 100% identical to the *sodA* gene sequence of EG005. The *sodA* gene sequence of EG005 differed by only one nucleotide from the *sodA* gene sequences of the eight closely related strains. As a result, the adenine (A) base corresponding to the 175th nucleotide on the *sodA* gene of EG005 was replaced by guanine (G) base in the other strains, and consequently, the amino acid sequence in EG005 contained threonine at this position, whereas the other strains had alanine. To investigate the evolutionary relationships among the different strains of *L. paracasei*, phylogenetic and ANI analyses were carried out using the 62 complete genome sequences of *L. paracasei*. According to the phylogenetic tree, *L. paracasei* strains were divided into several clusters, with EG005 positioned very close to CLP-C10, VHprobi OF10, and NJ strains. The short branch lengths, which indicated high genetic similarity (Figure 6A). In contrast, EG005 formed a close cluster with GM-080, LC2W, CBA3611, 1, VHProbi_F22, BD-II, TCS, and W56 strains in the ANI analysis, and the ANI values among these eight closest strains were observed to range from 99.66% to 99.69 (Figure 6B).

### 3.6 Overexpression of SOD in EG005

**Figure 7:**
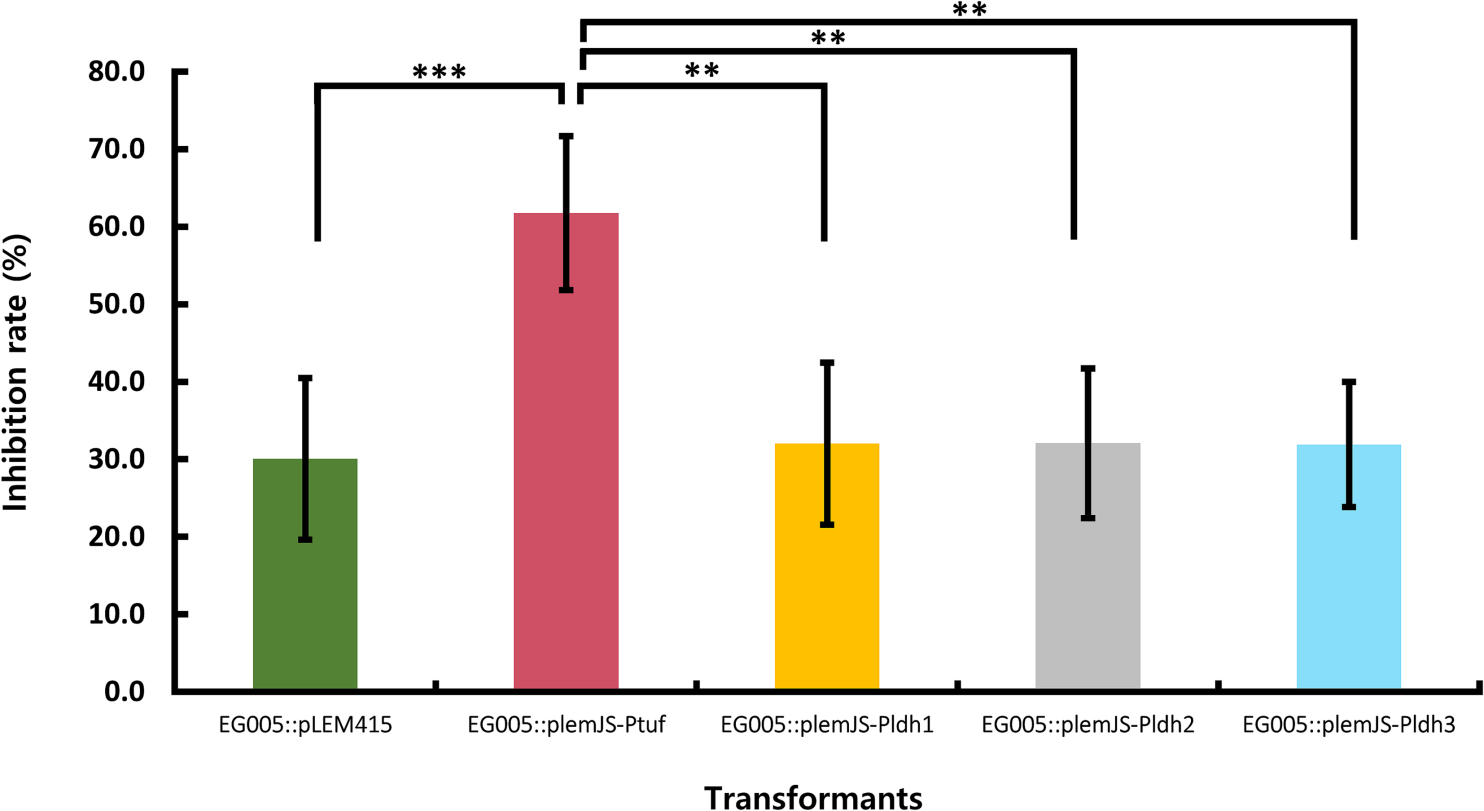
The result of SOD activity differences among recombinant strains. The EG005 harboring the pLEM415 vector was used as a control. The recombinant strain involving a *sodA* gene conjugated with P_tuf_ overexpression promoter exhibited the highest SOD activity. Error bars represent the standard deviation of three independent experiments. One-way ANOVA revealed a significant effect of strains on SOD activity. The *p*-value between all groups was shown on the top bar of the graph, and the p-value between P_tuf_ and the remaining groups is shown below it, respectively. (** *p* < 0.01, *** *p* < 0.001).

Four promoters (P_tuf_, P_ldh1_, P_ldh2_, P_ldh3_) known to be constitutive and highly active were selected to construct the SOD overexpression system in EG005. These promoters were present in the genome of EG005, and the sequence region was selected based on the presence of a TATA box and the Shine-Dalgarno sequence, a ribosomal binding site. All recombinants were constructed correctly as confirmed by restriction enzyme digestion and sequencing. The SOD activity of the EG005 recombinants harboring each strong promoter conjugated with *sodA* was compared to EG005 containing pLEM415, an empty plasmid vector, which was used as a control. As a result, the SOD activity of each EG005 recombinant harboring plemJS-P_ldh1_, plemJS-P_ldh2_, or plemJS-P_ldh3_ was only slightly improved compared to the SOD activity of EG005::pLEM415 (30.1%). The SOD activity values of ldh1, ldh2, and ldh3 were 32.0, 32.1, and 31.9%, respectively, with no significant differences observed between them and the empty vector. Therefore, the SOD activity among the remaining strains, except for EG005::plemJS-P_tuf_, was not statistically significant. On the other hand, EG005::plemJS-P_tuf_ showed SOD activity of 61.7%, which was nearly two-fold higher compared to EG005::pLEM415. In addition, it showed a much higher SOD activity than the other three EG005 recombinants harboring plemJS-P_ldh1_, plemJS-P_ldh2_, or plemJS-P_ldh3_ (Figure 7). To determine whether these results were statistically significant, a one-way ANOVA test was conducted and revealed a significant effect for strains on SOD activity (*p* = 0.00018). The *p*-values between EG005::plemJS-P_tuf_ and the remaining empty vector, plemJS-P_ldh1_, plemJS-P_ldh2_, and plemJS-P_ldh3_ were 0.00086, 0.00162, 0.00164, and 0.00156, respectively. These values were all very low and statistically significant, indicating substantial differences in SOD activity between strains.

## 4 Discussion

### 4.1 Antioxidant potential of EG005

The investigation into the SOD activity of EG005 under diverse pH conditions revealed that pH 7.0 is the optimal pH. At neutral pH, superoxide radicals remain stable and can interact most effectively with the SOD active site (Tuteja et al., 2015). Additionally, the amino acid residues composing EG005 SOD exhibit a suitable charge distribution at pH 7.0, contributing to high enzyme activity (Pinmanee et al., 2023). Specifically, Threonine, which contains a hydroxyl group (−OH), can form additional hydrogen bonds (Brockerman et al., 2014). This enhances the stability of the secondary structure of the protein, increasing its resistance to denaturation (Alber et al., 1987). In addition, Threonine’s polarity also boosts the stability of proteins across various pH ranges, allowing for better adaptability (Vassall et al., 2013). These findings may reflect natural selection during the evolution of EG005. As EG005 safely traverses through the acidic environment in the stomach and reaches the neutral pH of the small intestine, its enzyme activity will be optimized (Yamamura et al., 2023). Consequently, EG005 SOD has been shown to involve enough potential as an antioxidant, reducing oxidative stress in the gut and improving intestinal health by alleviating inflammation and maintaining microbial balance (Yasui and Baba, 2006; Dam et al., 2019).

### 4.2 Survival and stability of EG005

The properties of probiotics required to survive in various organs within the digestive system depend on their capacity to withstand and adapt to various environmental conditions, such as changes in pH, salinity, and temperature (Fiocco et al., 2020). Therefore, we evaluated the survival of EG005 under conditions of pH 2.5 and 0.3% bile salt, which closely mimic the environment of the human stomach and intestines (Castro-Lõpez et al., 2023). The human stomach typically maintains an acidity ranging from pH 1.5 to 3.5, while bile, produced in the liver and stored in the gallbladder, is released into the small intestine at concentration of 0.3 to 0.5% (Noriega et al., 2004). These conditions are valuable in predicting the survival likelihood of probiotics when consumed by humans. According to our research findings, EG005 demonstrates high acid and bile tolerance. Specifically, the survival rate recovered to approximately 100% after 3 h in the acid tolerance evaluation. The subsequent increase in survival rate, following an initial slight decrease, may be attributed to the gradual adaptation to acidic conditions through the regulation of membrane permeability or the activation of intracellular enzymes (Hassanzadazar et al., 2012; Wang et al., 2018). In the bile tolerance evaluation, EG005 maintained a survival rate above 80% for up to 6 h. Its high tolerance to acid and bile suggests that EG005 is well-suited to survive in the intestinal environment, making it a promising candidate for use in future probiotic therapies or functional foods Also, we used LGG as a control, since LGG is widely recognized for its well-established acid and bile tolerance characteristics (Corcoran et al., 2005), it is highly suitable as a control in experiments evaluating probiotics properties (Goldin, 1998; Segers and Lebeer, 2014).

### 4.3 Safety assessment of EG005

Hemolytic and antibiotic resistance tests are mainly performed to evaluate the safety of probiotics. Hemolysis is a virulence factor found in pathogens that destroys red blood cells, threatening the host’s life (Vesterlund et al., 2007). Therefore, it is essential that hemolytic traits are not found in Probiotic strains. In this context, EG005 was confirmed to be a safe strain, as it exhibited neither *β*-hemolytic nor *α*-hemolytic characteristics. Likewise, evaluating antibiotic resistance is another crucial step in assessing the safety of probiotics. A potential probiotic candidate strain must not exhibit resistance to antibiotics nor possess transposable antibiotic resistance (AR) genes (Gueimonde et al., 2013) because antibiotic resistance genes can be transferred through various mechanisms, including plasmids, transposons, bacteriophages, and horizontal gene transfer (HGT) (Bello-Lõpez et al., 2019). EG005 showed resistance only to kanamycin in the MIC test result, but no AR genes were detected from ABRicate analysis. Kanamycin resistance, however, is relatively common in most *lactobacilli* because of its intrinsic resistance to aminoglycosides (including kanamycin), due to features of their cell membranes that limit drug absorption (Campedelli et al., 2019; Lee et al., 2023). Consequently, this inherent resistance is not transferable from EG005 to other bacteria, indicating that EG005 is safe. The absence of hemolytic activity and antibiotic resistance further confirms EG005’s suitability as a probiotic strain.

### 4.4 Genomic features and functional annotations of EG005

We generated a new complete genome sequence of *L. paracasei* with a 99.5% BUSCO value. BUSCO is a bioinformatics tool used to assess the completeness of genome assemblies and annotations by searching for conserved single-copy orthologous (Simåo et al., 2015). A BUSCO score over 95% typically indicates that the high-quality assembly has been performed (Seppey et al., 2019). The annotation results of EG005 using KEGG and COG databases provide a good overview of the overall distribution of genetic and functional environments (Tatusov et al., 2000; Kanehisa et al., 2006). In our KEGG pathway analysis, CDSs involved in carbohydrate metabolism accounted for the largest proportion with 889 genes. Many of the genes involved in carbohydrate metabolism corresponded to glycolysis, gluconeogenesis, the TCA cycle, and various sugar transport systems (Mailloux et al., 2007). The presence of these genes highlights the ability of EG005 to perform both aerobic and anaerobic respiration (Shan et al., 2012). This flexibility can provide a competitive edge, enabling EG005 to thrive even in rapidly changing environments (Wu et al., 2023). Additionally, the multiple sugar transport system supports EG005’s excellent survival ability and environmental adaptability even in ecological niches with diverse carbohydrate availability (Liu et al., 2020). In the COG analysis the “Carbohydrate Transport and Metabolism (G)” category, which accounted for the highest percentage, further supports that EG005 has an excellent ability in carbohydrate metabolism (Ran et al., 2015). Next, the “Transcription (K)” category was notably high as well, which is crucial for managing stress responses by controlling gene expression (Namouchi et al., 2016). These functional annotations of EG005 highlight its evolutionary adaptation for optimizing energy production and regulatory mechanisms.

### 4.5 Phylogenetic relationships of EG005

Phylogenetic analysis was performed using CVTree and PYANI programs with whole genome sequences. Both programs were used to understand the evolutionary relationships by quantifying strain similarities (Pritchard et al., 2016). Using nucleotide sequences instead of amino acid sequences provides a comprehensive representation of the entire genome, including non-coding regions. Thus, whole-genome nucleotide data allows for the construction of detailed phylogenetic trees by leveraging the full genomic information (Xu and Hao, 2009). In our comparative phylogenetic tree analysis, however, CVTree and PYANI results showed different clustering among the same strains. This discrepancy arises because CVtree utilizes a composition vector (CV) approach that does not involve sequence alignment (Zuo and Hao, 2015), whereas, PYANI relies on pairwise nucleotide sequence similarities derived from sequence alignments performed by MUMmer and NUCmer (Richter and Rossellõ-Mõra, 2009). Consequently, these methodological differences may have resulted in the observed discrepancies between the two sets of results.

### 4.6 Promoter effects on overexpression of SOD in EG005

The P_tuf_ and P_ldh_ are widely known as promoters that induce high gene expression in Gram-positive bacteria such as *Lactobacillus* (Ma et al., 2018; Anbazhagan et al., 2013). The P_tuf_ regulates the gene for elongation factor Tu (EF-Tu), which plays an important role in transporting aminoacyl-tRNA to ribosomes during protein synthesis (Harvey et al., 2019). The P_ldh_ regulates the expression of lactate dehydrogenase (LDH), which is important in the lactic acid fermentation process (Romero et al., 2007). Based on the above, we constructed overexpression plasmids using a combination of one P_tuf_, three P_ldh_, and the *sodA* gene from EG005. These plasmids were then transformed into EG005. According to the result of the SOD activity test using these EG005 transformants, EG005 harboring plemJS-P_tuf_ exhibited the highest performance in inhibit ROS formation. This result suggests that the $P_{tuf}$ is effective in enhancing SOD activity in EG005. However, EG005 transformants harboring plemJS-P_ldh1_, plemJS-P_ldh2_, and plemJS-P_ldh3_ showed relatively lower SOD activity than EG005 harboring plemJS-P_tuf_. This result may be attributed to environmental effects on the expression level of each promoter. In fact, the *tuf* promoter is generally useful when stable and continuous expression is required and is often used to increase protein production (Henke et al., 2021; Kim et al., 2009). On the other hand, the *ldh* promoter originally promotes the expression of genes related to lactic acid fermentation, and its activity increases in fermentation processes such as low oxygen or high sugar concentration conditions (Song et al., 2017).

### 4.7 Potential and future directions for EG005

We confirmed that EG005, a strain with relatively high antioxidant activity, demonstrated probiotic properties and survival in simulated gastrointestinal conditions. Additionally, we performed *in silico* predictions to further assess its potential. Furthermore, we created an EG005 strain involving SOD overexpression and found that the P_tuf_ is effective in enhancing the SOD activity for EG005. These findings conclude that EG005, with SOD overexpression, has potential as an antioxidant to reduce oxidative stress in the gut and improve intestinal health by alleviating inflammation. However, further research is required to understand the realistic physiological effects *in vivo* by analyzing the impact of gene overexpression. In addition, as the stability of overexpression plasmid vectors over time cannot be guaranteed, the use of attB and attP-based recombination may offer a more reliable alternative for stable gene integration (Wu et al., 2021). This method would ensure continuous and stable expression of the *sodA* gene by facilitating site-specific recombination into the host genome (Lin et al., 2013). Further research should also focus on integrating this approach with CRISPR-Cas9 technologies to ensure long-term stability and effectiveness in commercial and medical applications (Ran et al., 2013; Doudna and Charpentier, 2014).

## Data Availability

The datasets presented in this study can be found in online repositories. The names of the repository/repositories and accession number(s) can be found in the article/Supplementary material.

## Footnotes

^1^https://github.com/rrwick/Porechop

^2^https://github.com/nanoporetech/medaka

^3^https://github.com/tseemann/abricate
