# Supplementary material for: Genomic insights and functional evaluation of Lacticaseibacillus paracasei EG005: a promising probiotic with enhanced antioxidant activity
Source: Front Microbiol. 2024 Oct 14;15:1477152. doi: 10.3389/fmicb.2024.1477152 (PMC11513463; doi:10.3389/fmicb.2024.1477152)
Supplement: Supplementary file 1 [file Table_1.DOCX]

Supplementary Material

# Supplementary Tables

**Table S1**. Primers used in this study for construction and amplification.

| **Target** | **Name** | **Restriction site** | **Sequence of primer 5’-3’** | **Amplicon size** |
| --- | --- | --- | --- | --- |
| P_tuf_ | F_ptuf | ApaⅠ | **GGGCCC**CGATACGTTTTCACTGTAAATGAAAGA AACTTG | 231 bp |
|  | R_ptuf | EcoRV, HindⅢ | **GATATC**TAT**AAGCTT**GAAAAAACGAACCTCCTGTATTTTTCGTAAG |  |
| P_ldh1_ | F_pldh1 | ApaⅠ | **GGGCCC**TATCTGATAGAGATGTCATGAAGCAGTCTG | 257 bp |
|  | R_pldh1 | EcoRV, HindⅢ | **GATATC**TAT**AAGCTT**GAAAACCCTTCCCCTCCACTC |  |
| P_ldh2_ | F_pldh2 | ApaⅠ | **GGGCCC**GAGGAACCACCTTTCTGGAAG | 302 bp |
|  | R_pldh2 | EcoRV, HindⅢ | **GATATC**TAT**AAGCTT**CTCAAATTCCTCCTCATGAAGATCTTG |  |
| P_ldh3_ | F_pldh3 | ApaⅠ | **GGGCCC**TGGAACTTTTATCCTCCTTGGGG | 683 bp |
|  | R_pldh3 | ClaⅠ, KpnⅠ | **ATCGAT**TAT**GGTACC**GGTGATATCATCCTTTCTTATGTGCATGC |  |
| *sodA* | F_sodA_HindⅢ | HindⅢ | TT**AAGCTT**ATGACATTTGTTTTGCCAGATTTACC | 634 bp |
|  | R_sodA_EcoRV | EcoRV | TT**GATATC**TCAGGCGTTTGTATCGGG |  |
|  | F_sodA_KpnⅠ | KpnⅠ | TT**GGTACC**ATGACATTTGTTTTGCCAGATTTACC |  |
|  | R_sodA_ClaⅠ | ClaⅠ | TT**ATCGAT**TCAGGCGTTTGTATCGGG |  |

**Table S2.** Plasmids used in this study.

| **Plasmids** | **Description** | **Reference/Source** |
| --- | --- | --- |
| pLEM415-ldhL-mRFP1 | *Lactobacillus-E.coli* shuttle vector, Erm ^R^, 7.3kb, f1 and ColE1 origin of replication | Bao et al |
| pGEM-T Easy | Subcloning vector, Amp^R^, 3.0kb, f1 and ColE1 origin of replication | Promega |
| pJS-$P_{\mathrm{tuf}}$ | P*_tuf_* promoter, Amp^R^, 3.2kb, added HindⅢ and EcoRV restriction enzyme site, f1 and ColE1 origin of replication | This study |
| pJS-$P_{ldh1}$ | P*_ldh1_* promoter, Amp^R^, 3.3kb, added HindⅢ and EcoRV restriction enzyme site, f1 and ColE1 origin of replication | This study |
| pJS-$P_{\mathrm{ldh}2}$ | P*_ldh2_* promoter, Amp^R^, 3.3kb, added HindⅢ and EcoRV restriction enzyme site, f1 and ColE1 origin of replication | This study |
| pJS-$P_{\mathrm{ldh}3}$ | P*_ldh3_* promoter, Amp^R^, 3.7kb, added KpnⅠ and ClaⅠ restriction enzyme site, f1 and ColE1 origin of replication | This study |
| pJS-S1 | *sodA* gene, Amp^R^, 3.6kb, added HindⅢ and EcoRV restriction enzyme site, f1 and ColE1 origin of replication | This study |
| pJS-S2 | *sodA* gene, Amp^R^, 3.6kb, added KpnⅠ and ClaⅠ restriction enzyme site, 7.1kb, f1 and ColE1 origin of replication | This study |
| pJS2-$P_{\mathrm{tuf}}$ | P*_tuf_* promoter and *sodA* gene, Amp^R^, 3.8kb, f1 and ColE1 origin of replication | This study |
| pJS2-$P_{ldh1}$ | P*_ldh1_* promoter and *sodA* gene, Amp^R^, 3.9kb, f1 and ColE1 origin of replication | This study |
| pJS2-$P_{\mathrm{ldh}2}$ | P*_ldh2_* promoter and *sodA* gene, Amp^R^, 3.9kb, f1 and ColE1 origin of replication | This study |
| pJS2-$P_{\mathrm{ldh}3}$ | P*_ldh3_* promoter and *sodA* gene, Amp^R^, 4.3kb, f1 and ColE1 origin of replication | This study |
| plemJS-$P_{\mathrm{tuf}}$ | pLEM415 containing P*_tuf_* promoter and *sodA* gene, Erm ^R^, 7.1kb, f1 and ColE1 origin of replication | This study |
| plemJS-$P_{ldh1}$ | pLEM415 containing P*_ldh1_* promoter and *sodA* gene, Erm ^R^, 7.2kb, f1 and ColE1 origin of replication | This study |
| plemJS-$P_{\mathrm{ldh}2}$ | pLEM415 containing P*_ldh2_* promoter and *sodA* gene, Erm ^R^, 7.2kb, f1 and ColE1 origin of replication | This study |
| plemJS-$P_{\mathrm{ldh}3}$ | pLEM415 containing P*_ldh3_* promoter and *sodA* gene, Erm ^R^, 7.6kb, f1 and ColE1 origin of replication | This study |
